# Contextual interpretation of COVID-19 pandemic among public space users in Ibadan Metropolis, Oyo State, Nigeria: An ethnographic review

**DOI:** 10.1371/journal.pone.0259631

**Published:** 2021-11-11

**Authors:** Mofeyisara Oluwatoyin Omobowale, Eniola Adetola Bamgboye, Akinfemi Akinyode, Olugbenga Samuel Falase, Taiwo Olabode Ladipo, Olufunmilayo Salami, Akindele Olupelumi Adebiyi

**Affiliations:** 1 Institute of Child Health, College of Medicine, University of Ibadan, Ibadan, Nigeria; 2 Department of Epidemiology and Medical Statistics, Faculty of Public Health, College of Medicine, University of Ibadan, Ibadan, Nigeria; 3 Oyo State Ministry of Health, Ibadan, Nigeria; 4 Department of Sociology, Lead City University, Ibadan, Nigeria; 5 Baylor College of Medicine, Houston, Texas, United States of America; 6 Department of Community Medicine, College of Medicine, University of Ibadan, Ibadan, Nigeria; University of Lincoln, UNITED KINGDOM

## Abstract

The COVID-19 pandemic has affected all dimensions of lives and has become a social problem as it continues to spread widely through the continuous interactions of people in public spaces where they earn a living. Curbing the spread of COVID-19 requires restrictions in these public spaces, however, the compliance to these measures depends largely on the understanding and interpretations of COVID 19 by users of these public spaces. This study examined the contextual interpretations of public space users about COVID-19 prevention in Ibadan Metropolis, Oyo State. The study was a rapid ethnographic survey in selected public spaces (markets and commercial motor parks) in Ibadan metropolis. Data were collected through participant observation, key informant interviews (3 females; 3 males) and in-depth interviews (30) with, traders, head porters, clients/buyers and commercial vehicle drivers in these public spaces. Interviews conducted were transcribed, sorted into themes using Atlas-ti 7.5.7 and subjected to interpretive-content analysis. Findings revealed that some respondents felt COVID-19 was brought into Nigeria by rich frequent global voyagers, others felt it was through “uncultured” sexual life or wrath of God. Some also doubted the existence of the disease and many of the respondents perceived COVID-19 as a disease reported by the government or a political propaganda to siphon funds. The users of the public spaces in Ibadan Metropolis have variegated perception about the existence and severity of this rapidly spreading virus and this has grave implications for COVID-19 control in the State. Thus, regular interaction with public space users are essential for control efforts.

## Background

The wide spread of the 2019 novel coronavirus (COVID-19) globally has affected humankind in different dimensions. The COVID-19 pandemic is challenging the very limit of societies, economies and relations across continents of the World, and Africa may be at the center of the economic decline [1]. Coronavirus disease (COVID-19) is an infectious disease caused by a newly discovered severe acute respiratory syndrome coronavirus 2 (SARS-CoV-2) [2].

Due to the enormous cases of mortality recorded by countries amid the COVID-19 pandemic in Asia, Europe, America and Africa, the World Health Organization (WHO) has been overwhelmed with efforts to curb the spread of the virus, successfully manage cases and ultimately, produce vaccines for future prevention [2]. Presently, there is no approved vaccines and medications certified by the World Health Organization to completely cure the virus; but there are public health preventive measures, such as, regular washing of hands, social distancing, use of face mask and avoidance of social gatherings [2].

Despite these measures, there have been an exponential increase in COVID-19 cases, as there were 5,956,503 confirmed cases and 367,403 confirmed deaths as at May 31, 2020 worldwide [3]. Thus, the pandemic has gone beyond the bio-medical explanation of germs, disease and health to include the social dimension, as it continues to spread widely through the continuous interactions of people in the world [4].

On the 27th of February 2020, Nigeria recorded the first case of COVID-19, in Lagos. By March 22 2020, Oyo State confirmed its first case of COVID-19. The continuous outbreak of the pandemic in some other cities, in the country made the Nigerian government to swiftly move to curb its deleterious effect by imposing ban on free movement, inter-state movement, social gatherings and all public functions of more than 20 people, including religious worships [5]. Supportively, some state governments including Oyo State also pronounced dawn to dusk curfews to limit the operations of clubs and night parties. In Oyo State, public and religious gathering with more than 20 people were banned; schools, offices and non-food markets non-pharmaceutical out-lets were closed down, only essential services like intra-state transportation, food markets and pharmaceutical out-fits were allowed to open, all done in order to curb the spread of COVID-19 [6].

Though these measures were aimed at containing the spread of the virus, some experts have shown that there are less expensive measures to curtail the spread of this virus which are largely behavioural and hinge on core values and morals [7]. These behavioural shared values are commonly presented in public spaces in most modern cities. These public spaces bind cities together and they include common assets owned, maintained and used by all members of the society [8–10].

Motor parks and market spaces are open and public spaces, where people interact closely, irrespective of ethnicity, gender and socio-economic status [11, 12]. These spaces also accommodate largely informal workers like commercial car drivers, tri- and motorcyclists, head porters, hawkers, market men and women [13–15], who rely solely on daily income as means of survival, hence, there is a great potential for the spread and conversely the containment of COVID-19 pandemic in such public spaces. Prevention of COVID-19 just like other influenza-like illnesses involves the use of behavior-oriented practices, such as handwashing, or policies on social distancing including recommendations to the avoidance of mass gathering which further impinge on the public spaces in most cities where confirmed cases are seen [16]. However, the compliance to the preventive measures laid down in unlocked public spaces has been a major concern as a result of the massive interaction of people in such spaces. The adherence to laid down policies depends largely on the primary understanding and interpretations of users of these public spaces about the COVID-19 pandemic. Hence, an in-depth understanding of the public space and its users will be beneficial to containment of COVID-19 in traditional states such as Oyo State. This study therefore examined the contextual interpretations of COVID 19 in public spaces in Ibadan, capital of Oyo State and one of the largest Cities in West Africa.

## Methodology

The study was a rapid ethnographic survey of COVID-19 and public spaces in Ibadan metropolis from 23, April 2020 to 21 May 2020. The city of Ibadan right from the pre-colonial era has been heterogeneous in population with a mixture of Yoruba, Igbo and Hausa-Fulani ethnic groups [17]. There had been a total of 190 confirmed cases of COVID-19 in Ibadan Metropolis as at the time of this study making it an epi center for Oyo State. The rapid ethnographic design was adopted to record emic and etic perspectives of the public to understand the socio-cultural context of COVID-19 within its natural social setting in Ibadan and the existing social interactional practices (close knit interactions versus social distancing). In this article, we present only the contextual interpretation of COVID-19 in these spaces.

Markets and commercial motor parks were selected, partly because they are spaces where many people of various classes and cultures mingle frequently and daily for several social and economic reasons. These are also spaces of “easy employment and a center of trading and melting pot of cultures” that give livelihood to many downtrodden in the city. The selected study sites were also based on the published map of COVID-19 in Oyo State. Data were collected through participant observation, photo-voices, six key informant interviews (3 females; 3 males) and thirty in-depth interviews with, traders, head porters, clients/buyers and commercial vehicle drivers in Ibadan.

During data collection period, the research team assumed the role of a temporary public space user, with the permission of leaders and individuals within the public spaces. Conversations, gossip, and rumors around COVID-19 and the larger society illuminated structures, beliefs, and social relations through sermons and admonitions from clerics and traditionalists of various other religions on the public spaces studied were used to gain insight into relationships, lived realities, and ideological strategies around COVID-19. Interviews were conducted in Yoruba and English, recorded in note pads and/or audio tape and later transcribed into English language, read, coded, sorted with themes with the aid of Atlas-ti 7.5.7. Subsequently, interpretive-content analysis was done. During data collection, the researchers protected themselves by wearing face masks, keeping social distance of 2 meters during interviews and practicing regular hand wash/ sanitizer use. All willing respondents were asked to wear nose masks and keep distance of 2 meters before the commencement of interviews. The study was approved by the Oyo State Ethical Review Board. Verbal informed consent were obtained from all participants before the commencement of interviews witnessed by either a representative of the leadership and/or at least two other users of the public space. Verbal informed consent and permission were also obtained from leaders and gate keepers of all selected public spaces studied. In case of minors, the adolescents were semi-emancipated, informed assents were obtained from them in addition to their parents/ and or guardians verbal informed consents and witnessed by representative of leadership. The process of all assents and consents were audio-recorded.

## Results

A total of six key informants, age range (36 to 88 years) were interviewed in six public spaces while thirty in-depth interviews (IDI) were conducted among users of these public spaces. The mean age of the IDI participants was 47.0 ± 18.6 years and majority of them were in the 45–64 years age group. IDI participants interviewed included traders (21), transporters (5), head porters (3) and an apprentice (1) (Tables 1 and 2).

**Table 1:** Socio-demographic of key informants interviews respondents.

Stakeholder	Gender	Occupation
Traders association	Male	Food Stuff
Female Market Leader	Female	Clothes Dealer
Food Stuff Sellers’ Association	Female	Food stuff seller
Female Market Leader	Female	Food items seller
Motor park Leader	Male	Commercial Motor Driver
Okada Riders Association	Male	Commercial Motorcycle Rider

**Table 2:** Age and sex distribution of in-depth interviewees.

Age group	Male	Female	Total
Less than 25	4(80.0)	1(20.0)	5(16.7)
25–44	4(57.1)	3(42.9)	7(23.3)
45–64	6(42.9)	8(57.1)	14(46.7)
65 years and above	1(25.0)	3(75.0)	4(13.3)
Total	15(50.0)	15(50.0)	30(100.0)

### Contextual interpretations of COVID 19 in Ibadan public space

All informants knew that first outbreak of COVID-19 was from China and had spread round the world. A few respondents understood and believed that COVID-19 is real, easily contagious, risky but preventable. The COVID-19 is popularly referred to as *arun Koro/ajakale arun aifojuri (Coro/* invisible epidemic disease) in Ibadan public space. The *arun Koro* is described as deadly, contagious and dangerous disease which originated from China, brought into Nigeria by rich frequent global voyagers- *aarun awon alakowaba (disease of those who can put others in trouble)*. Some also interpreted the deadly virus as *arun ato’hunriwa* (imported disease). More interestingly, it is also called *class leveler* disease which in their own meaning is interpreted as “disease that has denied the rich from running to overseas for better treatment”. This is evident in many responses as exemplified in the following statement: *“The thing that is making me happy is that the leaders will now realize that some people are suffering and need their attention*. *If you examine it well*, *nobody can go out like before*. *For instance*, *if it is a sickness that is affecting just an individual*, *they would have gone out of the country*, *so this did not allow them to understand the extent of suffering the masses are facing*. *I really think this Koro wants the leaders especially the ones that are ready to have a change of heart know that people are really suffering*, *imagine a lot of people that have been infected” (IDI/Male /Driver)*.

Another respondent added that: *“Now both the rich and the poor are now in the country*. *They cannot go abroad again*, *we all dey [sic] for this country and we dey [sic] same level now” (IDI/Male/Trader)*.

A significant number of the respondents interpret COVID-19 as the disease of the rich and the elite who can afford luxuries of life like travelling by air, eating exotic diet and riding of cars. The statement below represents the view of many respondents from all sites visited for this study:

*“Haaaa (she shook her head and twisted her fingers backward)*, *no I cannot get it*. *Because I am not walking in the shadow of corona*, *I don’t have money to travel out*. *I pray and I move away from crowd*, *I am careful*, *I don’t eat rich food*, *there is no money and corona has seen that I don’t eat well so it will not come near me at all” (IDI/Female/Head Porter)*.

Many of the respondents perceived COVID-19 as a disease reported by the government. They doubted the existence of the virus in Nigeria, and many still think it is mere political propaganda and has no real manifestation in Nigeria. This perception is reflected in statements like:

*“Ha… it is the government that said so; if they did not see it (winked eyes) they would not have said it*. *I have said it now*, *if the government did not see it they will not say it exists but personally I have not seen it and God will not allow me to see it*. *But they said it exists and it is not a joke*. *As they have said it exists*, *let us accept that it exists and what we are expected to do we should do just that” (IDI/Male/ Cab Driver)*

This was further corroborated by another informant that said: *“Actually*, *most people do not believe the virus exist*, *they say it is as if we are just being punished*, *they did not believe because it is not among the poor masses*. *If it was to be among the poor masses*, *they would have believed” (IDI/Male/Tricycle Rider)*.

A key informant further asserted thus: *“Hmmmm*, *according to what they are saying*, *we are hearing it exists but*, *we have not seen it around us*. *We did not even hear of it in our neighborhood*. *So*, *please help us beg the government to help us by lifting the lockdown for us people to go back to our various activities of livelihood” (KII/Male/Trader)*

And this also sealed the perception of the existence of the virus: *“No*, *I don’t believe it exists*. *I don’t believe in the existence of Coronavirus*. *Ehhn*, *the reason why I don’t believe in the existence is because some people*, *get involved in it because they want to have money and become rich and all kinds*. *Some people are suffering from the consequences of their sins*. *So*, *I don’t believe in the existence of Coronavirus*. *It is another food for the boys [sic]” (IDI/Female/Trader)*.

A significant number of informants categorically stated that they do not believe COVID-19 is real. They claimed it is to make those in government to further enrich their pockets. Besides, many other respondents perceived COVID-19 as the wrath of God sent to the World to punish the wrongdoings of mankind.

An informant stated that coronavirus is an incurable illness—*“We do not even know if it’s God’s anger that caused it” (IDI/Female/Trader)*. *Another key informant added that “we don’t know the source of this killer disease; it means God is talking to us*. *He is the God of mystery; we need to run back to him*. *He is the only one with the true cure; all these washing and staying far from people cannot help us*, *only God can” (KII/Male/Driver)*.

The study revealed that many respondents view that a major cause of the present pandemic is the test and/or punishment from God because of the overflowing sins and bloodshed of our leaders/wealthy class to help them rethink their ways and treat the masses right.

A trader emphatically mentioned that God is angry with us because of our sins. She explained thus: *“What is happening is God’s wrath on the people*. *It is a warning to call us back to Himself*. *All countries*, *companies*, *churches are now closed down*. *God wants to show how powerful He is to the whole world”*. *“**Tulasi le wo lu*, *ki Olorun ma mo si bi*?*”—meaning Can trouble enter the city and God will not know about it*?*- (IDI/Female /Trader)*.

Reacting to the effect of the COVID-19 and the lockdown in the state, another trader supported the above response and reaffirmed that God is angry. She reinforced her claim with a verse in the Bible that: *“Although there are several reasons that they said is the cause of coronavirus*, *but I want to tell you that it is God is very angry with us*. *Even all religious places have been shut down*. *In Isaiah 26*:*20*, *the Lord has warned us to shut our doors and go into our chambers until His anger will pass*. *And you know that the lockdown starts on March 26*, *2020*, *so you can see that God is giving us a powerful message*. *There is nothing to say again*, *God is telling us to seek for forgiveness and make atonement for our sins” (IDI/Female/Trader)*.

This was similarly expressed by another trader: *“I have not heard of the cause*, *but if God wants to test his people*, *he can use any means*. *I will say we should just move closer to God and serve God in the right and expected way*. *If not probable because our sins are so numerous*, *since the time I have been on this earth*, *I have never heard of a thing like this… punishment for sin*. *I pray that God will forgive us of our wrongs” (IDI/Male/Trader)*.

**Fig 1:**
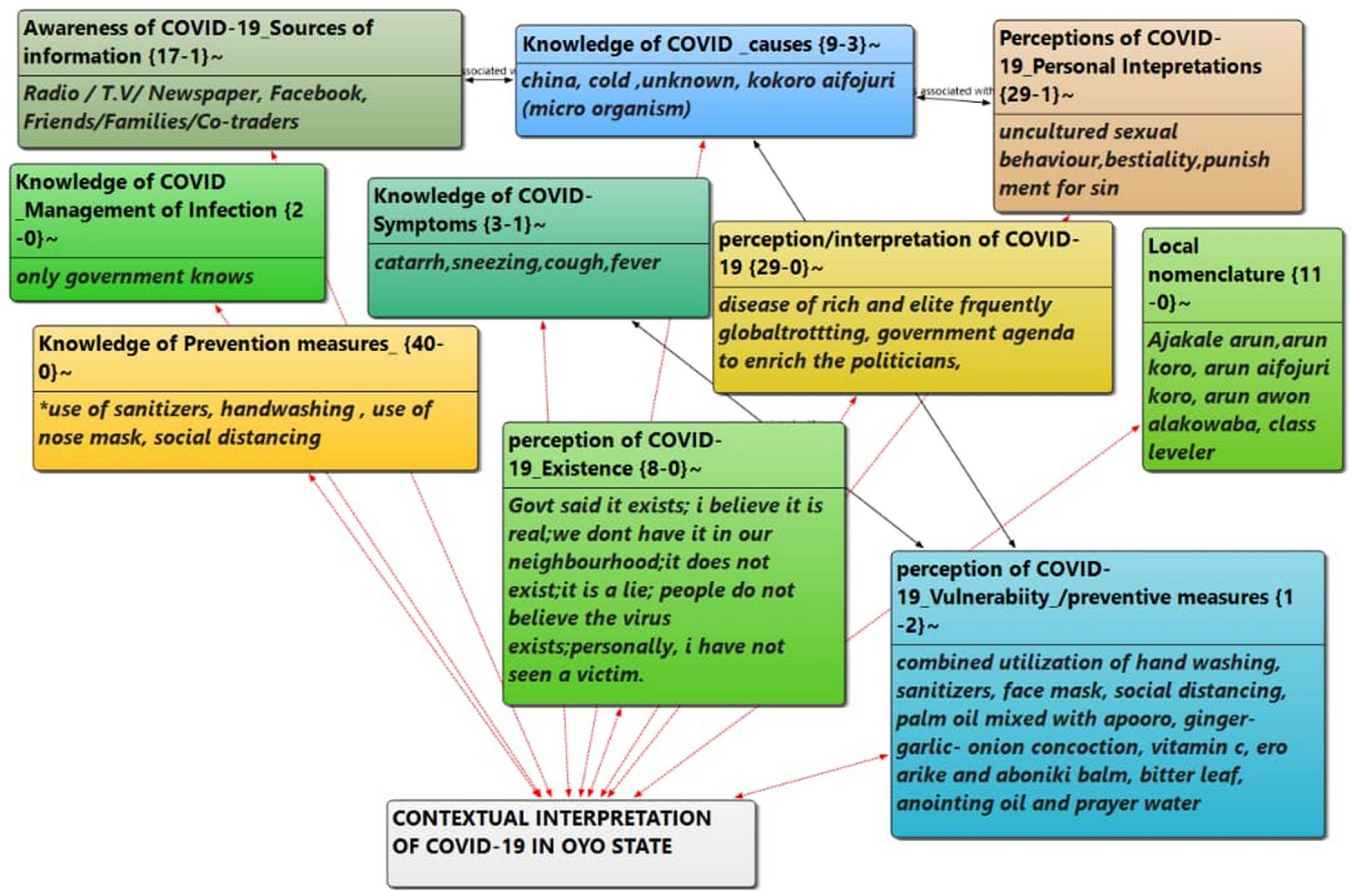
Network analysis of contextual interpretation of COVID-19 among respondents.

Fig 1 shows the network analysis of the contextual interpretation of COVID-19 among the users of public spaces in Ibadan Metropolis. This shows that many of the interpretations were interrelated.

## Discussion

The study revealed the existence of class biases that can negatively impact containment and prevention of COVID-19 in Ibadan and Oyo State at large. Some of the observed interpretations were based on emerging facts but the inferences were often wrong. For example, the notion of the disease being imported may have been based on the observation that the initial cases in Ibadan were seen among those who had considerable history of travel outside the country or exposure to those with such history. This is not peculiar to this pandemic as history has documented the spread of infectious diseases as being associated with international travel [18–20]. While some people believe that COVID-19 is an “elite disease”, some consider it to be a class leveler disease–a situation whereby the rich and poor are not equal, and which invariably sends a message to the elite that the masses are suffering. Although it is not true that the disease is an elite disease, but it gives the public space users and most informal workers some perceived relief that it is an avenue for the Nigerian governments to feel some of the pains that the masses are going through, particularly, when they visit the hospitals. With international travel restrictions occasioned by the pandemic, politicians and top government officials are trapped from travelling overseas for medical checkup [21]. Thus, it gives the public space users the belief of payback time, such that government officials will have to use the often-run-down hospitals and health centers. Despite having some form of information about the existence of COVID-19, some of the users of public spaces still doubt its existence. Some claimed that the victims of the virus have never been revealed by the government either through pictures and videos and that they have never come across anyone that has the virus. Others maintained that it was something that government said it is.

All these taken together, illustrates the greater level of suspicion with which occupiers of public spaces view the government with regards to public disclosure and consistency in leading by example [22].

Majority of public space users linked the cause of COVID-19 to religious factors. Many public space users believe that sins and other unpleasant activities that people engage in are the causes of coronavirus, particularly, the uncultured sexual life that people practice. Users of public spaces that believed that the emergence of COVID-19 was associated with sins of the world opined that lockdown time is for people to keep away from one another and ask for forgiveness. It is clear that such religious interpretations and references show the intricate link and role played by religion in African societies [23]. In addition, Roderick J. Lawrence in his piece on responding to COVID-19 posits that public adherence to guidelines cannot be assumed due to the influence of cultural, social and psychological factors [24].

The findings from our study emphasize the importance of societal context in understanding complex public health issues as it affects the society. Public spaces are owned by the society and provides a veritable platform for social cohesion and interactions. If utilized properly, public spaces could also be leveraged for the common good such as promoting a better understanding of the preventive guidelines for COVID-19 prevention. Thus, it is important that decision makers take bold steps to harness the understanding inherent in public spaces in planning appropriate interventions for COVID-19 control.
